# Deletion of *Rptor* in Preosteoblasts Reveals a Role for the Mammalian Target of Rapamycin Complex 1 (mTORC1) Complex in Dietary‐Induced Changes to Bone Mass and Glucose Homeostasis in Female Mice

**DOI:** 10.1002/jbm4.10486

**Published:** 2021-03-24

**Authors:** Pawanrat Tangseefa, Sally K. Martin, Agnieszka Arthur, Vasilios Panagopoulos, Amanda J. Page, Gary A. Wittert, Christopher G. Proud, Stephen Fitter, Andrew C.W. Zannettino

**Affiliations:** ^1^ Adelaide Medical School, Faculty of Health and Medical Science University of Adelaide Adelaide South Australia Australia; ^2^ Cancer Program, Precision Medicine Theme, South Australian Health and Medical Research Institute Adelaide South Australia Australia; ^3^ Nutrition, Diabetes & Gut Health Program, Lifelong Health Theme, South Australian Health and Medical Research Institute Adelaide South Australia Australia; ^4^ Freemasons Foundation Centre for Men's Health University of Adelaide Adelaide South Australia Australia; ^5^ School of Biological Sciences, University of Adelaide Adelaide South Australia Australia; ^6^ Central Adelaide Local Health Network Adelaide South Australia Australia

**Keywords:** BONE MARROW ADIPOSE TISSUE, DIET‐INDUCED INSULIN RESISTANCE, DIET‐INDUCED OBESITY, mTORC1, PREOSTEOBLAST

## Abstract

The mammalian target of rapamycin complex 1 (mTORC1) complex is the major nutrient sensor in mammalian cells that responds to amino acids, energy levels, growth factors, and hormones, such as insulin, to control anabolic and catabolic processes. We have recently shown that suppression of the mTORC1 complex in bone‐forming osteoblasts (OBs) improved glucose handling in male mice fed a normal or obesogenic diet. Mechanistically, this occurs, at least in part, by increasing OB insulin sensitivity leading to upregulation of glucose uptake and glycolysis. Given previously reported sex‐dependent differences observed upon antagonism of mTORC1 signaling, we investigated the metabolic and skeletal effects of genetic inactivation of preosteoblastic‐mTORC1 in female mice. Eight‐week‐old control diet (CD)‐fed *Rptor*_*ob*_^*−/−*^ mice had a low bone mass with a significant reduction in trabecular bone volume and trabecular number, reduced cortical bone thickness, and increased marrow adiposity. Despite no changes in body composition, CD‐fed *Rptor*_*ob*_^*−/−*^ mice exhibited significant lower fasting insulin and glucose levels and increased insulin sensitivity. Upon high‐fat diet (HFD) feeding, *Rptor*_*ob*_^*−/−*^ mice were resistant to a diet‐induced increase in whole‐body and total fat mass and protected from the development of diet‐induced insulin resistance. Notably, although 12 weeks of HFD increased marrow adiposity, with minimal changes in both trabecular and cortical bone in the female control mice, marrow adiposity was significantly reduced in HFD‐fed *Rptor*_*ob*_^*−/−*^ compared to both HFD‐fed control and CD‐fed *Rptor*_*ob*_^*−/−*^ mice. Collectively, our results demonstrate that mTORC1 function in preosteoblasts is crucial for skeletal development and skeletal regulation of glucose homeostasis in both male and female mice. Importantly, loss of mTORC1 function in OBs results in metabolic and physiological adaptations that mirror a caloric restriction phenotype (under CD) and protects against HFD‐induced obesity, associated insulin resistance, and marrow adiposity expansion. These results highlight the critical contribution of the skeleton in the regulation of whole‐body energy homeostasis. © 2021 The Authors. *JBMR Plus* published by Wiley Periodicals LLC on behalf of American Society for Bone and Mineral Research.

## Introduction

Dysregulated insulin signaling and the development of insulin insensitivity are common features of many chronic disorders including obesity, diabetes, and heart disease.^(^ ^1^ ^)^ Furthermore, excessive nutrient consumption, or overnutrition, is a major contributing factor to the increasing prevalence of these metabolic diseases in humans.^(^ ^2^ ^)^ At the center of cellular responses to nutritional challenges is the mammalian target of rapamycin complex 1 (mTORC1) complex, the primary‐nutrient‐sensing hub that responds to changes in intracellular nutrient status to promote cellular growth and anabolic metabolism. Binding of insulin to its receptor (INSR) indirectly activates mTORC1, which in turn promotes messenger RNA (mRNA) translation, ribosome biogenesis, and lipid synthesis, among other processes.^(^ ^3^ ^)^ mTORC1 negatively regulates insulin signaling by disrupting the interaction between INSR and its adaptor protein, IRS1, via (i) activation of ribosomal protein S6 kinase (S6K1), which phosphorylates IRS1 at Ser^270/307/1101^ and thus induces ubiquitin‐mediated proteasomal degradation of IRS1^(^ ^4^, ^5^, ^6^ ^)^; (ii) activation and stabilization of the receptor growth factor receptor‐bound protein 10 (Grb10), which inhibits IRS1 from binding to activated INSR^(^ ^7^, ^8^ ^)^; and/or (iii) direct phosphorylation of IRS1 at Ser^636/639^, sites that lie in close proximity to the phosphatidylinositol‐3′ kinase (PI3K) activation motif, the phosphorylation of which suppresses activation of PI3K downstream of IRS1.^(^ ^9^ ^)^

In addition to insulin, multiple nutrient signals independently stimulate the mTORC1 pathway (reviewed in Ali et al.^(^ ^10^ ^)^). These include amino acids, in particular the branched chain amino acid leucine (BCAAs), via Ras‐related small GTP binding protein (RAG) guanosine triphosphatase (GTPase)‐dependent signaling.^(^ ^11^ ^)^ mTORC1 activity is also stimulated by glucose and fatty acids through cellular energy status. Under conditions of low cellular energy levels (high adenosine monophosphate [AMP]/adenosine triphosphate [ATP] ratio) the AMP‐dependent kinase (AMPK) represses mTORC1 signaling through phosphorylation of the tuberous sclerosis 1/2 (TSC1/2) complex, a negative regulator of mTORC1^(^ ^12^ ^)^ and RAPTOR.^(^ ^13^ ^)^ Consequently, in states of nutrient overload, mTORC1 is chronically activated, leading to sustained stimulation of S6K1 and S6K1‐mediated phosphorylation and degradation of IRS1.^(^ ^14^ ^)^ This desensitizes cellular insulin signaling and impairs the PI3K/protein kinase B (AKT) signaling pathway, thus rendering cells resistant to insulin.^(^ ^15^ ^)^ Thus, as a consequence of chronically high nutrient levels, mTORC1 remains active and maintains the negative feedback loop to the INSR, leading to systemic insulin insensitivity.^(^ ^16^ ^)^

Inhibition of mTORC1 or elements of the mTORC1 signaling pathway, either genetically or pharmacologically, is associated with an improved metabolic profile. For example, treatment with rapamycin, an mTORC1 inhibitor, dose‐dependently increased lifespan in both male and female mice.^(^ ^17^ ^)^ However, when relative life‐extension was compared, the effects were consistently greater in the females than males.^(^ ^18^ ^)^ Furthermore, whole‐body inactivation of S6K1, a downstream target of mTORC1, protects against diet‐induced insulin resistance and obesity, due to elevated lipolysis and metabolic rate, in male mice only,^(^ ^19^ ^)^ but extends lifespan in female mice.^(^ ^20^ ^)^ Consistent with this, increased expression of eukaryotic translation initiation factor 4E binding protein 1 (4E‐BP1), whose inhibitory effect on mRNA translation is countered by mTORC1, protects against both aging‐induced and high‐fat diet (HFD)‐induced obesity and metabolic impairments only in male mice,^(^ ^21^ ^)^ whereas combined disruption of 4E‐BP1 and 4E‐BP2 has the opposite effect, with these mice displaying increased adiposity and sensitivity to both HFD‐induced obesity and insulin resistance.^(^ ^22^ ^)^ Collectively, these results suggest that the beneficial effects of reduced mTORC1 signaling is sex‐dependent.

The skeleton has recently emerged as a critical insulin target tissue. Importantly, insulin signaling in osteoblasts (OBs), is necessary for whole‐body glucose homeostasis, whereby OB‐specific deletion of INSR (INSR^OB−/−^) causes elevated blood glucose, increased insulin resistance, and increased peripheral fat mass in animals fed a normal diet,^(^ ^23^, ^24^ ^)^ while overexpression or downregulation of INSR in OBs results in profound changes in systemic glucose metabolism in response to an obesogenic diet.^(^ ^25^, ^26^ ^)^ Consistent with these observations, we recently showed that suppression of the mTORC1 complex in pre‐OB improved glucose handling in male mice fed a normal or HFD, at least in part, through an increase in OB insulin sensitivity leading to upregulation of glucose uptake and glycolysis.^(^ ^27^ ^)^ Given the sexually dimorphic effects observed upon reduced mTORC1 signaling in other studies, we investigated the effects of genetic inactivation of preosteoblastic mTORC1 (OB‐mTORC1) in female mice.

## Materials and Methods

### Transgenic mice and diet

All mice were bred and group‐housed (maximum 5 mice/cage) in pathogen‐free conditions at the South Australian Health and Medical Research Institute (SAHMRI) Bioresources Facility (Adelaide, SA, Australia) under a 12‐hour light–dark cycle (lights on at 7:00 a.m.) at constant temperature (20–23°C), with ad libitum access to a standard chow diet (Teklad Global Diet #2918: 18.6% protein, 6.2% fat; Envigo, Indianapolis, IN, USA) and water. For diet‐induced obesity studies, mice were fed an HFD (Specialty Feeds #SF16‐096: 19.4% protein, 23% fat [43.4% of total energy from fat]; Specialty Feeds, Perth, Australia) from weaning to 16 weeks of age. All studies were performed with institutional ethics approval (SAHMRI Animal Ethics Committee, #SAM164). Female conditional knockout mice, in which *Rptor* was disrupted in early osteoprogenitor cells, were generated using *Osx1‐GFP::Cre* mice,^(^ ^28^ ^)^ R26eYFP mice,^(^ ^29^ ^)^ and *Rptor* ^*fl/fl*^ mice^(^ ^30^ ^)^ as described.^(^ ^31^ ^)^ Wild‐type littermates were used as controls as detailed in our previous study.^(^ ^27^ ^)^ Animals were weighed twice‐weekly and at the end of the study, body length and whole‐body lean and fat mass were measured postmortem using a dedicated mouse dual X‐ray absorptiometer (Lunar Piximus II; GE Medical Systems, Madison, WI, USA) or EchoMRI Body Composition Analysis (EchoMRI LLC, Houston, TX, USA).

### Metabolic phenotyping

Insulin and glucose tolerance tests (ITT and GTT, respectively) and indirect calorimetry assessment were performed as described.^(^ ^27^ ^)^

### High‐resolution micro–computed tomography scanning

X‐ray micro–computed tomography (μCT) was performed using a SkyScan 1176 (Bruker, Kontich, Belgium). Tibias were scanned at 55 kV/455 mA using a 0.5‐mm aluminum filter, a 0.6 rotation step, and three‐frame averaging with an isometric resolution of 8.78 μm/pixel. Three‐dimensional reconstruction of the scan data (smoothing, 1, ring artifact, 8, and beam hardening, 30%) was performed using NRecon (Bruker). Analysis of the bone microarchitecture was performed using CTAn (Bruker). Cortical bone measurements were calculated as the average of measurements in the lateral, medial, caudal, and cranial orientation from three slices of cortical bone. Analysis and nomenclature of μCT follow recommended standard.^(^ ^32^ ^)^ Specifically, slices were selected in relation to the primary spongiosa. For analysis of the proximal tibia, a trabecular region of interest was manually defined to exclude the cortex. For trabecular bone, the region of analysis was determined as an area of 150 microtomographic slices, commencing 35 slices distal to the primary spongiosa of a 13‐mm tibia. Images were reconstructed and threshold values established using a specimen‐specific threshold. To ensure that the analysis of trabecular bone was conducted in corresponding regions of the secondary spongiosa proximal to the growth plate, the distance from the primary spongiosa and the area analyzed was normalized to the length of the tibia. Tibial length measurements were performed using CTAn software (Bruker) and taken from the proximal tibial head to the fibular notch.

### Enzyme‐linked immunosorbent assay

Fasted blood samples were collected via cardiac puncture into Minicollect tubes (Greiner Bio One, Kremsmünster, Austria) as described.^(^ ^27^ ^)^ Commercial enzyme‐linked immunosorbent assay (ELISA) kits were used for the measurement of: insulin (EZRMI‐13K; Millipore, Burlington, MA, USA), leptin (EZML‐82K; Millipore), adiponectin (EZMADP‐60K; Millipore), osteocalcin [OCN] (BT‐470; Alfa Aesar, Lancashire, UK), and undercarboxylated OCN (unOCN) (MK129; TaKaRa Bio, Otsu, Japan) per the manufacturer's instructions.

### Histology

Tibias were fixed in 10% formalin and decalcified in 0.5M ethylenediaminetetraacetic acid (EDTA) pH 8.0 solution before being processed and embedded into paraffin wax. Sections (5 μm) were stained with hematoxylin and eosin, and images were taken using a NanoZoomer 2.0 (Hamamatsu Photonics, Hamamatsu City, Japan). The region of interest for the proximal mouse tibias was defined as a region of secondary spongiosa distal from the primary spongiosa to a distance of 1.8 mm. The percentage adipocyte area per marrow area (Ad.Ar/Ma.Ar), adipocyte area per number of adipocytes (Ad.Ar/Ad.N), and number of adipocytes per marrow area (N.AdC/Ma.Ar) were quantified using Osteomeasure software (OsteoMetrics, Decatur, GA, USA). Bone histomorphometry nomenclature follows recommended standards.^(^ ^33^ ^)^

### Protein isolation and Western blotting

Protein isolation from tissue samples, Western blotting, and quantitative analysis were performed as described.^(^ ^27^ ^)^ Antibodies for immunoblotting are phospho‐rpS6^Ser240/244^ (CST2215S), phosphor‐AKT^Ser473^ (CST4060), ACTIN (A5441), and UCP1 (ab209483).

### RNA isolation and quantitative reverse‐transcription polymerase chain reaction

RNA isolation of tissue samples and quantitative reverse‐transcription polymerase chain reaction (RT‐PCR) were performed as described.^(^ ^27^ ^)^ The following forward and reverse primer pairs were used for real‐time PCR: *Actin*, 5′‐TTGCTGACACGATGCAGGA‐3′ and 5′‐AAGGGTGTAAAACGCAGCTC‐3′; *Pcg‐1α*, 5′‐TGTGTGCTGTGTGTCAGAGT‐3′ and 5′‐ACCAGAGCAGCACACTCTATG‐3′, and *Ucp1*, 5′‐TGGTGAACCCGACAACTTCC‐3′ and 5′‐ GGCCTTCACCTTGGATCTGAA‐3′.

### Statistical analysis

All data are presented as presented as box‐plots with mean, maximum, and minimum values. All data were plotted. Statistical analyses were performed using a one‐way or two‐way analysis of variance (ANOVA) with a Tukey's post hoc test or an unpaired Student's *t* test using GraphPad Prism (GraphPad Software, Inc., La Jolla, CA, USA). Significance was accepted at *p* < 0.05, with asterisks denoting *p* value levels: *p < 0.05; **p < 0.01; ***p < 0.001 unless stated otherwise.

## Results

### Loss of mTORC1 function in pre‐OBs reduced bone mass but increased systemic insulin sensitivity in female mice

In previous studies, we have investigated the function of mTORC1 in skeletal development, osteoblast‐mediated B cell development, and skeletal control of glucose homeostasis using male mice with conditional deletion of *Rptor*, an essential component of mTORC1, in pre‐OBs (*Rptor*_*ob*_^*−/−*^ mice).^(^ ^27^, ^31^, ^34^ ^)^ In our most recent study, we showed that suppression of the mTORC1 complex in OBs improved glucose handling in male mice fed a control diet (CD) or high‐fat diet (HFD), at least in part, by increasing OB insulin sensitivity leading to upregulation of glucose uptake and glycolysis.^(^ ^27^ ^)^ To determine if this important observation was sex‐dependent, female *Rptor*_*ob*_^*−/−*^ mice were fed a CD and assessments of bone phenotype and glucose metabolism were examined.

**Fig. 1:**
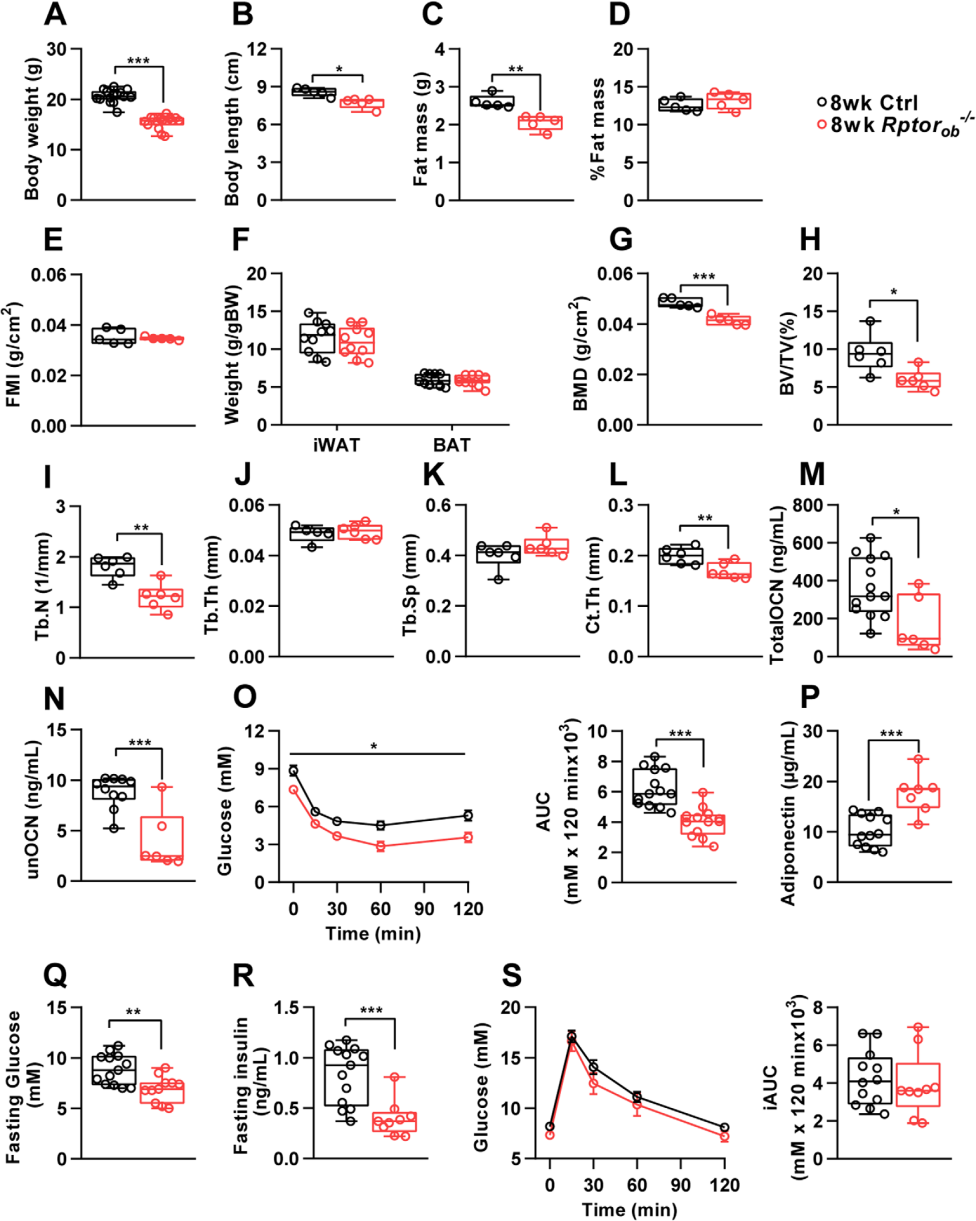
Loss of OB‐mTORC1 function in 8‐week‐old female mice results in low bone mass but increased peripheral insulin sensitivity. (*A*) Body weight (*n* = 15/genotype). (*B*) Body length, as measured from nose to anus (*n* = 5/genotype). (*C*) DXA analysis of fat mass and (*D*) % fat mass normalized to total body weight (*n* = 5/genotype). (*E*) FMI (*n* = 5/genotype). (*F*) Body weight‐adjusted iWAT and gWAT mass (*n* = 10/genotype). (*G*) Whole‐body BMD, measured by DXA (*n* = 5/genotype). (*H*–*L*) Quantitative assessment of the trabecular bone of the tibia measured by high‐resolution μCT: BV, TV, Tb.N, Tb.Th, Tb.Sp, Ct.Th (*n* = 6/genotype). (*M*) Serum levels of total OCN (*n* = 6–13/genotype). (*N*) Serum levels of unOCN (*n* = 6–10/genotype). (*O*) Insulin tolerance test and area under the curve analysis at 8 weeks of age (*n* = 11–13/genotype). (*P*) Serum adiponectin levels (*n* = 6–10/genotype). (*Q*) Fasting blood glucose levels (*n* = 11–13/genotype). (*R*) Fasting serum insulin levels (*n* = 9–13/genotype). (*S*) Glucose tolerance test and incremental area under the curve analysis (*n* = 11–13/genotype). In all panels, data are presented as box‐plot with mean, maximum, and minimum values. *p < 0.05, **p < 0.01, ***p < 0.001, Student *t* test. Abbreviations: μCT, micro–computed tomography; BMD, bone mineral density; BV, bone volume; Ct.Th, cortical thickness; DXA, dual‐energy absorptiometry; FMI, fat mass index; gWAT, gonadal white adipose tissue; iWAT, inguinal white adipose tissue; mTORC1, mammalian target of rapamycin complex 1; OB, osteoblast; OCN, osteocalcin; Tb.N, trabecular number; Tb.Th, trabecular thickness; Tb.Sp, trabecular separation; TV, total volume; unOCN, undercarboxylated OCN.

As shown in Figure 1*A* and *B*, 8‐week‐old female *Rptor*_*ob*_^*−/−*^ mice weighed significantly less and were approximately 10% shorter in length than the controls. However, although total fat mass was significantly lower in female *Rptor*_*ob*_^*−/−*^ mice (Figure 1*C*), correcting fat mass to account for the differences in total body weight (as a percentage of total body weight: Figure 1*D*) or body length (fat mass index [FMI]: fat mass/height^2^, Figure 1*E*) showed that the reduction in fat mass in the females was proportional to their lower body mass. Consistent with this, no significant differences in body weight–adjusted fat depots were observed in female *Rptor*_*ob*_^*−/−*^ mice relative to controls (Figure 1*F*). Conversely, bone mineral density (BMD; g/cm^2^) measured by whole‐body dual‐energy X‐ray absorptiometry (DXA) scan, was significantly reduced in the female *Rptor*_*ob*_^*−/−*^ mice (Figure 1*G*). Furthermore, analysis of trabecular bone, using μCT, revealed a significant reduction in bone volume (BV/TV) and trabecular number (Tb.N) in the female *Rptor*_*ob*_^*−/−*^ mice compared to controls, whereas both trabecular thickness (Tb.Th) and separation (Tb.Sp) were unchanged (Figure 1*H*–*K*). Cortical thickness (Ct.Th) was also significantly reduced in the tibial diaphysis of female *Rptor*_*ob*_^*−/−*^ mice (Figure 1*L*). Consistent with their low bone mass, circulating levels of both total and undercarboxylated osteocalcin were significantly reduced in the *Rptor*_*ob*_^*−/−*^ mice (Figure 1*M*,*N*). Interestingly, despite no change in their percentage of whole‐body adiposity, female *Rptor*_*ob*_^*−/−*^ mice showed improved whole‐body insulin sensitivity, as measured by an ITT, which was associated with elevated circulating adiponectin levels (Figure 1*O*,*P*). Moreover, female *Rptor*_*ob*_^*−/−*^ mice have significantly lower fasting glucose and insulin levels (Figure 1*Q*,*R*). Conversely, no significant change in glucose tolerance was observed in female *Rptor*_*ob*_^*−/−*^ mice (Figure 1*S*).

### Suppression of OB‐mTORC1 protects female mice from diet‐induced insulin resistance

**Fig. 2:**
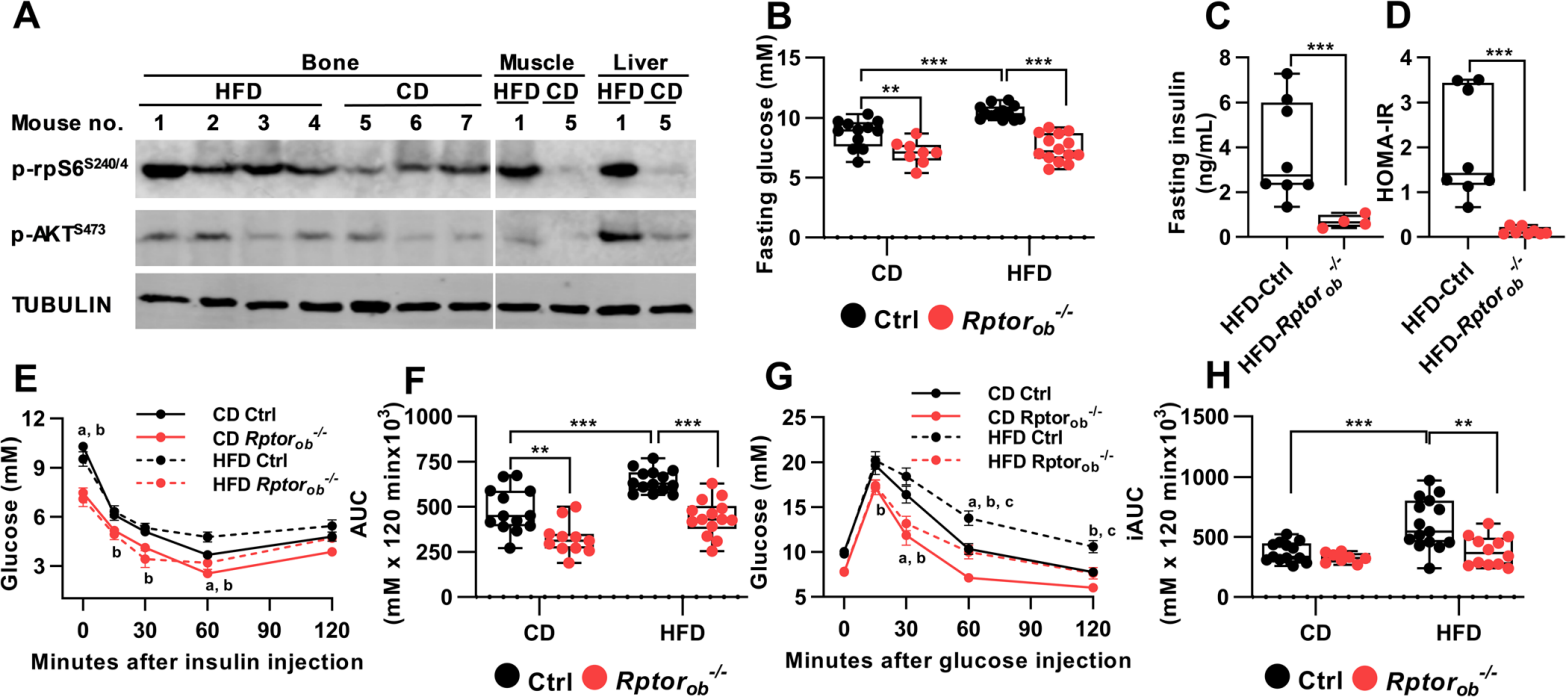
Female HFD‐fed *Rptor*_*ob*_^*−/−*^ mice are protected against diet‐induced impairment of glucose metabolism. (*A*) Wild‐type mice were fed an HFD or CD for 12 weeks and steady‐state phosphorylation levels of rpS6 and AKT in indicated tissues were measured by Western blotting. (*B*) Fasting blood glucose levels in 16‐week‐old (*n* = 8–14/genotype). (*C*) Fasting serum insulin levels at 16 weeks of age (*n* = 8/genotype). (*D*) HOMA‐IR calculation for 16‐week old HFD‐fed mice (*n* = 8/genotype). (*E*) Insulin tolerance test and (*F*) area under the curve analysis at 16 weeks of age (*n* = 6–15/group). (*G*) Glucose tolerance test and (*H*) incremental area under the curve analysis (*n* = 8–15/genotype). All panels except *A*, *E*, and *G*: Data are presented as box‐plot with mean, maximum, and minimum values. *p < 0.05, **p < 0.01, ***p < 0.001, two‐way ANOVA with Tukey's post hoc test. For panel *E* and *G*: Data are expressed as mean ± SEM. ^a^ *p* < 0.05 between CD‐fed Ctrl and CD‐fed *Rptor*_*ob*_^*−/−*^, ^b^ *p* < 0.05 between HFD‐fed Ctrl and HFD‐fed *Rptor*_*ob*_^*−/−*^and ^c^ *p* < 0.05 between CD‐fed Ctrl and HFD‐fed Ctrl, two‐way ANOVA with Tukey's post hoc test. Abbreviations: AKT, protein kinase B; ANOVA, analysis of variance; CD, control diet; Ctrl, control; HFD, high‐fat diet; HOMA‐IR, homeostatic model assessment of insulin resistance; rpS6, ribosomal protein S6; SEM, standard error of the mean.

A previous study by Wei et al.^(^ ^25^ ^)^ showed that bones from wild‐type mice that have been challenged with a HFD exhibit features of insulin resistance such as reduced insulin‐stimulated phosphorylation of AKT at Ser^473^ and downregulation of the INSR. This reduction in OB insulin signaling was also observed in other primary insulin target tissues such as liver, skeletal muscle, and adipose tissues.^(^ ^25^ ^)^ To investigate if insulin resistance in bone is associated with hyperactivation of mTORC1, we fed wild‐type mice an obesogenic HFD for 12 weeks (from 4 to 16 weeks of age) and measured the basal phosphorylation levels of ribosomal S6 (rpS6 Ser^240/244^), a substrate for p70S6K, a primary mTORC1 effector,^(^ ^35^ ^)^ as a readout of mTORC1 activity. As a control, we measured basal levels of AKT P‐Ser^473^, which has previously been shown to be increased in liver and muscle in response to HFD feeding.^(^ ^36^, ^37^, ^38^ ^)^ Consistent with these previous studies, basal levels of AKT P‐Ser^473^ were also elevated in bone (Figure 2*A*). Moreover, basal levels of rpS6 P‐Ser^240/244^ were higher in bone after HFD feeding, compared to CD, suggesting that hyperactivation of mTORC1 could be involved in the development of bone‐specific insulin resistance.

To determine if suppression of OB‐mTORC1 under HFD feeding could protect animals from developing diet‐induced glucose intolerance and insulin resistance, age‐matched female control and *Rptor*_*ob*_^*−/−*^ mice were fed a CD or an obesogenic HFD for 12 weeks and changes to metabolic parameters were assessed. Fasting glucose levels in CD‐fed and HFD‐fed *Rptor*_*ob*_^*−/−*^ mice were significantly lower than the control mice fed a matching diet (Figure 2*B*). In HFD‐fed *Rptor*_*ob*_^*−/−*^ mice, this was accompanied by significantly lower fasting insulin levels (Figure 2*C*). Calculation of the homeostatic model assessment of insulin resistance (HOMA‐IR) identified *Rptor*_*ob*_^*−/−*^ mice as being more insulin‐sensitive than HFD‐fed control mice (Figure 2*D*). Consistent with this, ITT revealed a higher responsiveness to insulin in *Rptor*_*ob*_^*−/−*^ mice compared to the controls, irrespective of diet (Figure 2*E*,*F*). Importantly, although no difference in glucose tolerance was observed in the CD‐fed *Rptor*_*ob*_^*−/−*^ mice compared to the CD‐fed controls (consistent with CD‐fed *Rptor*_*ob*_^*−/−*^ mice at 8 weeks; Figure 1*S*), HFD‐fed *Rptor*_*ob*_^*−/−*^ mice were protected from developing glucose intolerance as evidenced by significant lower incremental area under the curve calculation (iAUC) compared to HFD‐fed control mice (Figure 2*G*,*H*). Collectively, these results suggest that suppression of OB‐mTORC1 protects female mice from developing diet‐induced systemic impairment of glucose metabolism.

### Female *Rptor*_*ob*_^*−/−*^ mice are resistant to an HFD‐induced increase in fat mass

**Fig. 3:**
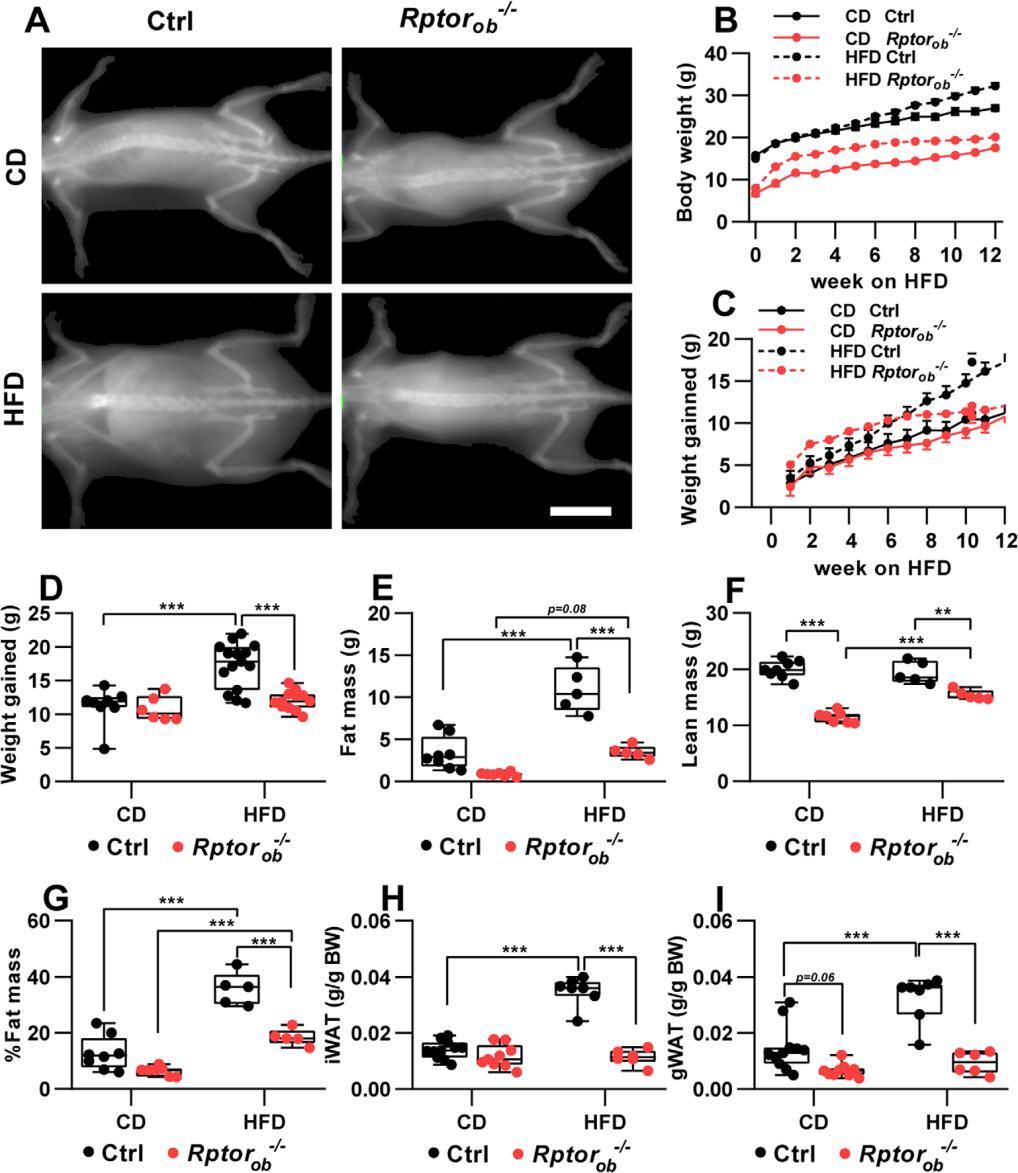
Female HFD‐fed *Rptor*_*ob*_^*−/−*^ mice are protected against diet‐induced obesity and total fat gain. (*A*) Representative DXA images of 16‐week‐old mice. Scale bar = 2 cm. (*B*) Temporal body weight changes (*n* = 6–15/genotype). (*C*) Temporal and (*D*) end‐of‐study (16 weeks old) body weight gain in response to CD or HFD (*n* = 6–15/genotype). (*E*–*G*) DXA analysis of total fat and lean mass and % fat mass normalized to total body weight (*n* = 6–9/genotype). (*H*–*I*) iWAT and gWAT fat pad mass, normalized to body weight (*n* = 6–9/genotype). All panels except *A*: Data are presented as box‐plot with mean, maximum, and minimum values. **p < 0.01, ***p < 0.001, two‐way ANOVA with Tukey's post hoc test. Abbreviations: ANOVA, analysis of variance; CD, control diet; DXA, dual‐energy absorptiometry; gWAT, gonadal white adipose tissue; HFD, high‐fat diet; iWAT, inguinal white adipose tissue.

Analysis of body weight over time revealed diet‐specific and genotype‐specific differences in weight gain. After 12 weeks on the HFD, control (Ctrl) mice weighed significantly more and showed substantial weight gain relative to CD‐fed controls (17.26 ± 1.02 g in HFD‐Ctrl vs. 11.21 ± 1.01 g in CD‐Ctrl: Figure 3*A*–*D*). Conversely, *Rptor*_*ob*_^*−/−*^ mice gained the same amount of weight on both diets (12.05 ± 0.44 g in HFD‐*Rptor*_*ob*_^*−/−*^ vs. 10.80 ± 0.79 g in CD‐*Rptor*_*ob*_^*−/−*^; Figure 3*B*,*C*). Notably, analysis of weekly weight gain showed that, although HFD‐fed *Rptor*_*ob*_^*−/−*^ mice initially gained weight more rapidly, compared to HFD‐fed controls, weight gain in the *Rptor*_*ob*_^*−/−*^ mice plateaued after approximately 6 weeks (Figure 3*C*,*D*). Thus, *Rptor*_*ob*_^*−/−*^ mice gained only a small amount of additional weight thereafter (i.e., from 6 to 13 weeks on HFD, control mice gained 7.2 g, whereas *Rptor*_*ob*_^*−/−*^ mice gained only 1.7 g; Figure 3*D*). Further examination of end‐point body composition revealed that HFD‐fed control mice mainly gained fat (with no difference in lean mass observed between CD‐fed Ctrl and HFD‐fed Ctrl, whereas HFD‐fed *Rptor*_*ob*_^*−/−*^ mice gained both fat and lean mass relative to CD‐fed *Rptor*_*ob*_^*−/−*^ mice (Figure 3*E* and *F*, respectively). Consistent with this, the percentage of fat mass, relative to body weight, was significantly higher in control mice relative to *Rptor*_*ob*_^*−/−*^ mice on both diets (Figure 3*G*). Furthermore, analysis of inguinal and gonadal fat depots revealed significantly lower body weight–adjusted fat mass in the HFD‐fed *Rptor*_*ob*_^*−/−*^ mice compared to the HFD‐fed control mice (Figure 3*H*,*I*).

### Female *Rptor*_*ob*_^*−/−*^ mice display increased expression of UCP1 in the inguinal fat despite no significant changes in total energy expenditure

**Fig. 4:**
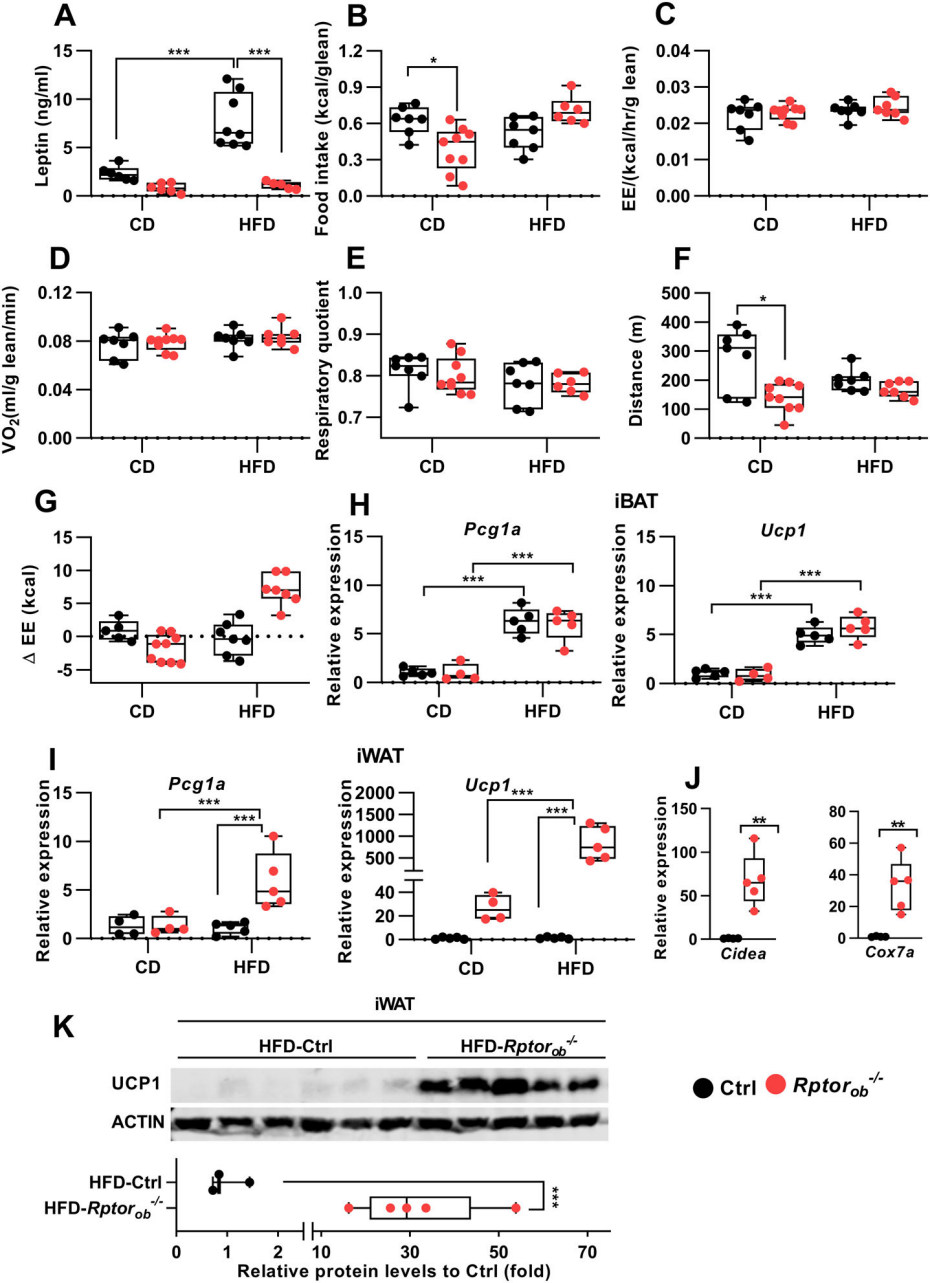
Female HFD‐fed *Rptor*_*ob*_^*−/−*^ mice exhibit upregulated UCP1 expression despite unchanged daily food intake, oxygen consumption, and energy expenditure. (*A*) Serum leptin levels in HFD‐fed mice at 16 weeks of age (*n* = 6–8/genotype). (*B*) Daily food intake normalized to lean mass (kcal/g lean mass) in CD‐fed or HFD‐fed mice (*n* = 7–9/genotype). (*C*) Energy expenditure, normalized to lean mass, (*n* = 7–9/genotype). (*D*) Volume of oxygen consumption normalized to lean mass, (*n* = 7–9/genotype). (*E*) Average respiratory quotient (*n* = 7–9/genotype). (*F*) Total activity measured as distance of movement (*n* = 7–9/genotype). (*G*) Energy balance calculated as energy expenditure‐energy intake (*n* = 7–9/genotype). Gene expression levels of BAT markers in CD‐fed and HFD‐fed iBAT (*H*) and iWAT (*I*), normalized to β‐actin (*n* = 5/genotype). (*J*) Gene expression levels of BAT markers in HFD‐fed iWAT. (*K*) Levels of UCP1 protein expression in HFD‐fed iWAT tissue (*top panel*) and quantitative analysis of protein levels relative to β‐actin (*bottom panel*) (*n* = 5–6/genotype). All panels except *K* (*top panel*): Data are presented as box‐plot with mean, maximum, and minimum values. *p < 0.05, ***p < 0.001, two‐way ANOVA with Tukey's post hoc test. Abbreviations: ANOVA, analysis of variance; BAT, brown adipose tissue; CD, control diet; HFD, high‐fat diet; iBAT, interscapular brown adipose tissue; iWAT, inguinal white adipose tissue.

To examine the underlying mechanisms responsible for the altered body composition of *Rptor*_*ob*_^*−/−*^ mice, serum leptin levels, food intake, energy expenditure, and physical activity were examined. To account for the differences in their body size, each of these metabolic parameters was normalized to lean mass. In keeping with an increase in fat mass (Figure 3*E*), circulating leptin levels were found to be significantly increased in the HFD‐fed control mice (Figure 4*A*). In contrast, despite a significant increase in %fat mass in HFD‐fed *Rptor*_*ob*_^*−/−*^ mice relative to CD‐fed *Rptor*_*ob*_^*−/−*^ mice (Figure 3*G*), no difference in leptin levels was observed (Figure 4*A*). Food consumption, normalized to lean mass, was lower in the CD‐fed *Rptor*_*ob*_^*−/−*^ mice compared to CD‐fed controls (Figure 4*B*). However, when fed an HFD, no significant difference in food intake was observed in *Rptor*_*ob*_^*−/−*^ mice relative to HFD‐fed controls (Figure 4*B*). The obesity‐resistant phenotype of *Rptor*_*ob*_^*−/−*^ mice was also independent of changes in total energy expenditure, oxygen consumption, respiratory quotient, or physical activity (Figure 4*C*–*F*). Of note, a significant reduction in physical activity was observed in the CD‐fed *Rptor*_*ob*_^*−/−*^ mice compared to the controls (Figure 4*H*). Interestingly, analysis of energy balance (i.e., energy intake minus energy expenditure) revealed a negative energy balance in CD‐fed *Rptor*_*ob*_^*−/−*^ mice and a positive energy balance in HFD‐fed *Rptor*_*ob*_^*−/−*^ mice (Figure 4*G*).

Previous studies have shown that a positive energy balance can induce uncoupled thermogenesis.^(^ ^39^, ^40^, ^41^ ^)^ To investigate this question, we examined expression of *Pcg1α* and *Ucp1* mRNAs in the interscapular brown adipose tissue (iBAT) of these mice. The mRNA levels of both *Pcg1α* and *Ucp1* were significantly upregulated in response to HFD; however, there was no significant difference between genotypes (Figure 4*H*). However, expression levels of *Pgc1a* and *Ucp1* in inguinal white adipose tissue (iWAT) were greatly upregulated in HFD‐fed *Rptor*_*ob*_^*−/−*^ mice (Figure 4*I*). Furthermore, a strong upregulation of the expression of other genes associated with thermogenesis (e.g., *Cidea* and *Cox7a*) was observed in HFD‐fed *Rptor*_*ob*_^*−/−*^ iWAT samples compared to HFD‐fed controls (Figure 4*J*). Western blotting of protein lysates made from iWAT revealed a significant increase in UCP1 protein levels in HFD‐fed *Rptor*_*ob*_^*−/−*^ mice compared to the HFD‐controls (Figure 4*K*).

### HFD feeding rescues defects in bone of female *Rptor*_*ob*_^*−/−*^ mice

Bone mass and skeletal integrity are greatly impacted by obesity and HFD feeding. Several lines of evidence demonstrate that increased marrow adiposity is associated with lower BMD and increased skeletal fragility in HFD‐fed rodents^(^ ^42^, ^43^ ^)^ and obese or diabetic human patients.^(^ ^44^, ^45^ ^)^ Importantly, a decrease in the number of differentiated OBs and diminished quantities of osteoid, leading to a poorly organized bone structure and decreased bone quality, and increased fracture risk are often observed in type 2 diabetic patients.^(^ ^46^, ^47^, ^48^ ^)^ To investigate the effects of diet and loss of OB‐mTORC1 function on skeletal development, trabecular and cortical bone in the secondary spongiosa proximal to the tibial growth plate of the 16‐week‐old control and *Rptor*_*ob*_^*−/−*^ female mice were analyzed. Female *Rptor*_*ob*_^*−/−*^ mice were found to have significantly shorter tibias than the controls, irrespective of diet (Table 1). Despite the significant anabolic role of mTORC1, no significant difference in BV/TV was observed in *Rptor*_*ob*_^*−/−*^ mice, relative to control mice, on either diet (Table 1) although a trend toward a decrease in BV/TV (*p* = 0.09) was observed in the HFD‐fed *Rptor*_*ob*_^*−/−*^ compared to the HFD‐fed controls (Table 1). The effect of HFD on trabecular bone in the control mice appeared to be minor because only trabecular bone pattern factor (Tb.Pf), which indicates levels of connectivity between trabecular structures, was significantly decreased in the HFD‐fed compared to CD‐fed controls (Table 1). Conversely, Tb.Th was significantly increased in the HFD‐fed compared to CD‐fed *Rptor*_*ob*_^*−/−*^ mice (Table 1). A significant decrease in Tb.N was observed in HFD‐fed *Rptor*_*ob*_^*−/−*^ mice compared to HFD‐fed controls (Table 1). Measurement of Ct.Th, at the proximal tibia, was significantly reduced in the *Rptor*_*ob*_^*−/−*^ mice compared to the controls in both diets (Table 1). However, analysis of cortical bone at the mid‐shaft or diaphysis of the tibia, which is composed of more inert cortical bone surrounding the intramedullary cavity, showed no significant changes in Ct.Th in *Rptor*_*ob*_^*−/−*^ mice compared to the controls in both diets (Table 1). Consistent with the proximal trabecular bone, HFD feeding did not have a substantial impact on midshaft cortical bone of the control mice (i.e., no significant changes in any parameters observed). Marrow diameter (Ma.Dm) and area (Ma.Ar) were significantly higher in the HFD‐fed compared to CD‐fed *Rptor*_*ob*_^*−/−*^ mice (Table 1). These increases in Ma.Dm and Ma.Ar in the HFD‐fed *Rptor*_*ob*_^*−/−*^ mice were associated with increase in bone area (B.Ar) and total bone area. Interestingly, although B.Ar and Ma.Ar showed a significant or a trend toward significant decrease in the CD‐fed *Rptor*_*ob*_^*−/−*^ mice compared to CD‐fed controls, no significant changes were observed between genotypes under HFD (Table 1).

**Table 1:** Trabecular and cortical bone properties in 16‐week‐old female mice fed a CD or HFD

Bone properties	CD‐Ctrl	CD‐*Rptor*_*ob*_^*−/−*^	HFD‐Ctrl	HFD‐*Rptor*_*ob*_^*−/−*^
Tibia length (mm)	14.453 ± 0.085	13.360 ± 0.324*	14.720 ± 0.155	13.715 ± 0.314**
Proximal tibia				
Trabecular bone				
BV/TV(%)	8.386 ± 0.462	7.885 ± 0.559	8.879 ± 0.255	7.271 ± 0.465
Tb.Th (mm)	0.059 ± 0.001	0.056 ± 0.001	0.061 ± 0.001	0.063 ± 0.001***
Tb.N (1/mm)	1.434 ± 0.082	1.399 ± 0.102	1.466 ± 0.034	1.146 ± 0.073**
Tb.Pf (1/mm)	32.262 ± 0.457	30.382 ± 1.103	28.564 ± 1.272*	28.519 ± 0.543
Tb.Sp (mm)	0.439 ± 0.015	0.408 ± 0.026	0.448 ± 0.006	0.461 ± 0.022
Cortical bone				
Ct.Th (mm)	0.156 ± 0.006	0.105 ± 0.005*	0.152 ± 0.006	0.125 ± 0.006** ^,^ ***
Midshaft tibia				
Ct.Th (mm)	0.294 ± 0.012	0.248 ± 0.015	0.286 ± 0.017	0.276 ± 0.011
Ma.Dm (mm)	0.793 ± 0.026	0.691 ± 0.026	0.876 ± 0.030	0.837 ± 0.033***
B.Ar (mm^2^)	0.823 ± 0.034	0.600 ± 0.053*	0.866 ± 0.053	0.800 ± 0.051***
Ma.Ar (mm^2^)	0.434 ± 0.019	0.326 ± 0.021	0.515 ± 0.032	0.497 ± 0.036***
Total bone area (mm^2^)	1.256 ± 0.050	0.926 ± 0.072*	1.381 ± 0.083	1.297 ± 0.085***

*Note*: Data are expressed as mean ± SEM from *n* = 5–7/genotype; two‐way ANOVA with Tukey's post hoc test.

Abbreviations: ANOVA, analysis of variance; B.Ar, bone area; BV/TV, bone volume/total volume; CD, control diet; Ctrl, control; Ct.Th, cortical thickness; HFD, high‐fat diet; Ma.Ar, marrow area; Ma.Dm, marrow diameter; SEM, standard error of the mean; Tb.Pf, trabecular bone pattern factor; Tb.N, trabecular number; Tb.Sp, trabecular separation; Tb.Th, trabecular thickness.

* *p* < 0.05 versus CD‐fed Ctrl group.

** *p* < 0.05 versus HFD‐fed Ctrl group;

*** *p* < 0.05 versus CD‐fed *Rptor*_*ob*_^*−/−*^ group.

### Decreased bone marrow adiposity observed in HFD‐fed *Rptor*_*ob*_^*−/−*^ mice

**Fig. 5:**
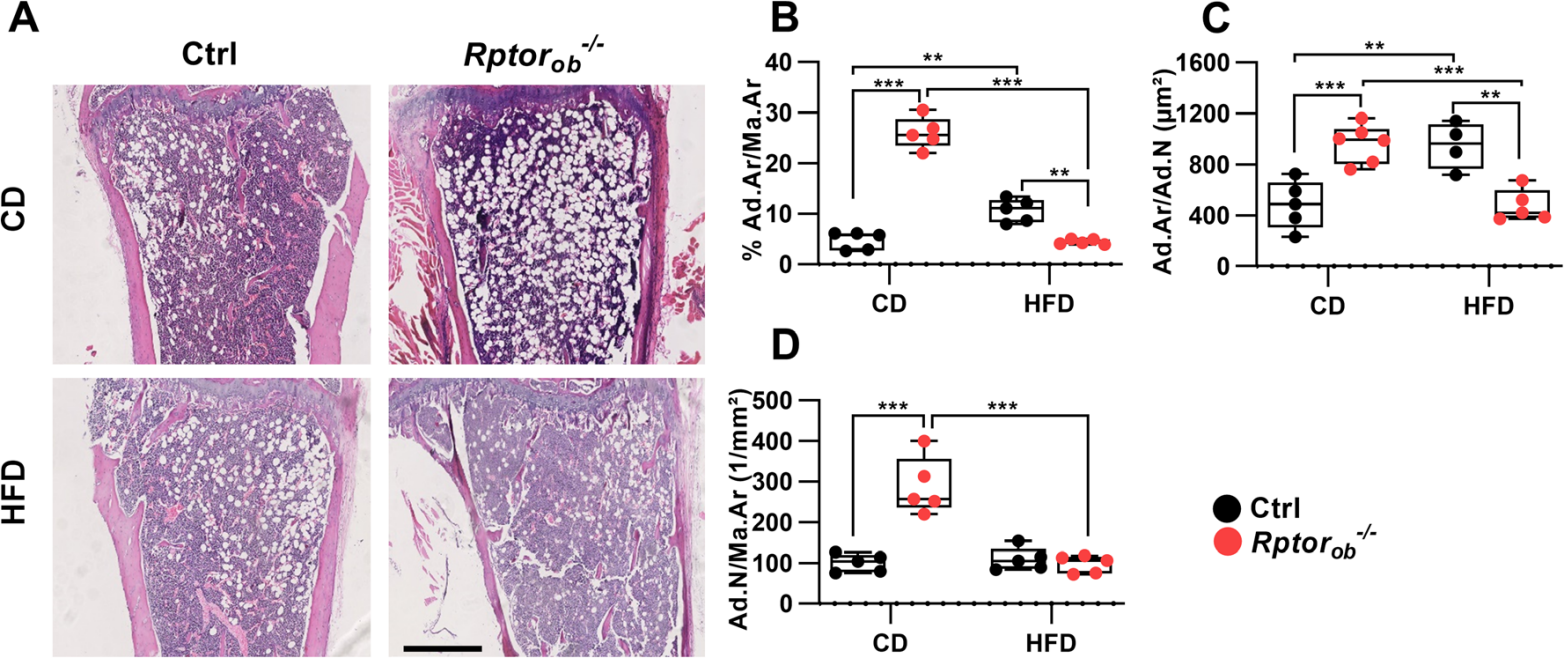
Female HFD‐fed *Rptor*_*ob*_^*−/−*^ mice are protected against HFD‐induced marrow adipose tissue expansion. (*A*) Representative H&E–stained sections of the proximal tibia in CD‐fed or HFD‐fed mice. Scale bar = 500 μm. (*B*) Percentage area occupied by adipocytes per marrow area (%Ad.Ar/Ma.Ar). (*C*) Average adipocyte size (Ad.Ar/Ad.N). (*D*) Numbers of adipocytes per marrow area (Ad.N/Ma.Ar). All panels except *A*: Data are presented as box‐plot with mean, maximum, and minimum values from *n* = 5/genotype. **p < 0.01, ***p < 0.001, two‐way ANOVA with Tukey's post hoc test. Abbreviations: Ad.Ar, adipocyte area; Ad.N, number of adipocytes; ANOVA, analysis of variance; CD, control diet; H&E, hematoxylin and eosin; HFD, high‐fat diet; Ma.Ar, marrow area.

To evaluate the effect of HFD on bone marrow adiposity, histomorphometric analysis of hematoxylin and eosin–stained sections of bones were performed. In addition to reduced cortical thickness, significantly higher levels of intramedullary adipocytes (i.e., percentage of area occupied by adipocytes per total marrow area (proximal tibia adiposity: %Ad.Ar/Ma.Ar), numbers of adipocytes per marrow area (N.Ad/Ma.Ar) and average adipocyte size (Ad.Ar/N.Ad) were observed in the tibias of CD‐fed *Rptor*_*ob*_^*−/−*^ mice compared to the CD‐fed controls (Figure 5*A*–*D*). Consistent with previous studies,^(^ ^43^, ^49^, ^50^ ^)^ 12 weeks of HFD feeding led to a significant increase in proximal tibia adiposity and larger average adipocyte size in the controls (Figure 5*B*,*C*). However, no significant changes in the number of adipocytes per area was observed, suggesting that HFD feeding results in hypertrophy but not hyperplasia of marrow adipocytes (Figure 5*D*). Intriguingly, marrow adiposity was significantly lower in the HFD‐fed *Rptor*_*ob*_^*−/−*^ mice relative to either CD‐fed *Rptor*_*ob*_^*−/−*^ mice or HFD‐fed controls (Figure 5*B*). This reduced in adiposity was associated with a significant decrease in both number and average size of adipocytes per marrow area relative to CD‐fed *Rptor*_*ob*_^*−/−*^ mice (Figure 5*C*,*D*).

## Discussion

In this study, we investigated the effects of bone‐specific inhibition of mTORC1 on the skeleton and metabolic parameters in response to a control or obesogenic HFD in female mice. Deletion of *Rptor*, an essential component of mTORC1, in pre‐OBs was shown to have a profound effect on skeletal development in young (8‐week‐old) mice, as evidenced by a significant reduction in trabecular bone volume and trabecular number; however, most of these differences were resolved by 16 weeks of age. Notably, although 12 weeks of HFD increased marrow adiposity with minimal changes in both trabecular and cortical bone in the female control mice, marrow adiposity was significantly reduced in HFD‐fed *Rptor*_*ob*_^*−/−*^ compared to both HFD‐fed control and CD‐fed *Rptor*_*ob*_^*−/−*^ mice. Furthermore, *Rptor*_*ob*_^*−/−*^ mice were resistant to HFD‐induced weight gain and protected from the development of diet‐induced insulin resistance.

### Bone phenotypes

Previous studies from our laboratory and others have shown that mTORC1 plays an important role in bone formation and OB differentiation, with conditional inactivation of OB‐mTORC1 resulting in severe osteopenia and increased skeletal fragility in male mice.^(^ ^31^, ^51^, ^52^ ^)^ In the current study, it was found that at 8 weeks of age, female *Rptor*_*ob*_^*−/−*^ mice not only exhibit a low bone mass phenotype (i.e., significantly reduced %BV/TV, decreased Tb.N in proximal tibia and reduced Ct.Th), but also display a marked whole‐body metabolic phenotype characterized by low glycemic levels, and increased insulin sensitivity. These findings are consistent with our previous observations in the male *Rptor*_*ob*_^*−/−*^ mice^(^ ^27^, ^31^ ^)^ (A comparison of phenotypes observed between male and female *Rptor*_*ob*_^*−/−*^ mice is summarized in Table 2). Unexpectedly, however, the trabecular bone defects observed in 8‐week‐old CD‐fed female *Rptor*_*ob*_^*−/−*^ mice were almost completely resolved by 16 weeks of age, although the reduction in cortical bone thickness persisted. This “normalization” of the trabecular bone defects could indicate an initial delay in trabecular bone formation in young *Rptor*_*ob*_^*−/−*^ mice that is resolved over time. Alternatively, female *Rptor*_*ob*_^*−/−*^ mice are protected from further age‐related bone loss such that the age‐induced decrease in trabecular BV/TV observed in the control mice reduced the magnitude of differences between control and *Rptor*_*ob*_^*−/−*^ mice. In support of this, studies have reported that, on standard chow diet, wild‐type females lose more bone and strength as they age,^(^ ^53^ ^)^ whereas bone structure and function were preserved in the chow‐fed wild‐type males up to 18 months of age.^(^ ^54^ ^)^

**Table 2:** Summary of phenotypes associated with loss of mTORC1 function in preosteoblasts in male and female *Rptor*_*ob*_^*−/−*^ mice fed normal chow and HFD

	CD‐fed		HFD‐fed, 16 weeks	
Phenotype	Female, 8 weeks old	Male, 4 and 12 weeks old	Female	Male
Weight gain			Constant after 6 weeks on HFD	
Body weight	↓	↓	↓	↓
% Fat	↔	↓	↓	↓
% Lean	↔	↑	↑	↑
BMD (g/cm^2^)	↓	↓	↔	↓
BV/TV (%)	↓	↓	↔	n/a
Tb.N (1/mm)	↓	↓	↓	n/a
Tb.Th (mm)	↔	↓	↔	n/a
Ct.Th (mm)	↓	↓	↔	n/a
iWAT (g/g BW)	↓	↔	↓	↓
gWAT (g/g BW)	↓	↓	↓	↓
BAT (g/g BW)	↔	↔	↓	↔
EE (kcal/g lean mass)	↔	↑	↔	↑ dark
Food intake (g/g BW)	↓	↔	↔	↔
RQ	↔	↓	↓ light↑ dark	↓ light ↑ dark
%Fecal lipid	↔	↔	↔	↔
Browning of WAT	n/a	n/a	↑	↑
Metabolic phenotype				
Fasting glucose	↓	↓	↓	↓
Insulin sensitivity	↑	↑	↑	↑
Glucose tolerance	↔	↑	↑	↑
GSIS	↑	↑	↑	↑
Serum analysis				
Fasting insulin	↓	↓	↓	↓
Leptin	↓	↓	↓	↓
Adiponectin	↑	↑	↑	↑
Total OCN	↓	↓	↓	↔
unOCN	↓	↓	↔	↔

*Note*: CD‐fed 4‐week‐old and 12‐week‐old *Rptor*_*ob*_^*−/−*^ mice^(^ ^31^ ^)^ and HFD‐fed 16‐week‐old *Rptor*_*ob*_^*−/−*^ mice.^(^ ^27^ ^)^

Abbreviations: ↑, increased; ↓, decreased; ↔, unchanged relative to control group for each sex and diet; BAT, brown adipose tissue; BMD, bone mineral density; BV/TV, bone volume/total volume; BW, body weight; CD, control diet; Ct.Th, cortical thickness; EE, energy expenditure; GSIS, glucose‐stimulated insulin secretion; gWAT, gonadal white adipose tissue; HFD, high‐fat diet; iWAT, inguinal white adipose tissue; mTORC1, mammalian target of rapamycin complex 1; n/a, data not available; OCN, osteocalcin; RQ, respiratory quotient; Tb.N, trabecular number; Tb.Th, trabecular thickness; unOCN, undercarboxylated osteocalcin; WAT, white adipose tissue.

In response to HFD feeding, we observed little or no significant changes in both trabecular and cortical bone parameters in the control mice. This was consistent with some^(^ ^43^, ^55^ ^)^ but not other studies.^(^ ^49^, ^50^, ^56^, ^57^, ^58^, ^59^, ^60^ ^)^ Studies using C57BL/6J male mice fed with HFD from 4 to 23 weeks of age,^(^ ^56^ ^)^ 12 to 23 weeks of age,^(^ ^57^ ^)^ or 7 to 28 weeks of age^(^ ^58^ ^)^ have reported positive correlation between obesity and BMD, associated with higher femoral Tb.Th and mineral content and higher cortical bone area and thickness despite lower bone formation and high marrow adiposity. Conversely, other studies stated deleterious effects in the distal femur and proximal tibia including lower trabecular BV/TV, increased bone turnover, and higher receptor activator of nuclear factor kappa B ligand (RANKL), tumor necrosis factor (TNF), and peroxisome proliferator–activated receptor (PPAR)‐gamma along with higher marrow adiposity and lower fracture resistance.^(^ ^49^, ^50^, ^59^, ^60^ ^)^ We speculate that the disparity between our female control mice and those from other studies could stem from the differences in animal strains, diet composition, and duration of HFD.

In contrast, for *Rptor*_*ob*_^*−/−*^ mice, HFD feeding resulted in significantly higher trabecular and cortical bone thickness (although trabecular number showed a trend toward reduction) in the proximal tibia compared to the CD‐fed mice. A previous study reported a correlation between trabecular thickness and systemic glycemic levels.^(^ ^61^ ^)^ Therefore, it is possible that some of the bone phenotypes, observed in the HFD‐fed *Rptor*_*ob*_^*−/−*^ mice, are due, in part, to the protective metabolic phenotypes of deleting *Rptor* in bone (i.e., maintain normal glucose homeostasis and resistance to HFD‐induced weight gain).

### Inactivation of OB‐specific nutrient sensing mTORC1 results in dietary restriction–like phenotypes

At 16 weeks of age, bone marrow adipose tissue (BMAT) was significantly elevated in the CD‐fed *Rptor*_*ob*_^*−/−*^ mice compared to the CD‐fed controls and conversely, BMAT was significantly reduced in HFD‐fed *Rptor*_*ob*_^*−/−*^ mice compared to both CD‐fed *Rptor*_*ob*_^*−/−*^ and HFD‐fed controls. These results were somewhat unexpected, given the consistent association between HFD feeding and enhanced marrow adiposity reported in previous animal studies.^(^ ^43^, ^49^, ^50^, ^57^, ^60^, ^62^ ^)^ It is interesting to note that, unlike male *Rptor*_*ob*_^*−/−*^ mice, elevated BMAT was not observed in 4‐week‐old female *Rptor*_*ob*_^*−/−*^ mice (i.e., the age at which mice were assigned to either CD or HFD; Supplementary Figure S1). This suggests that the BMAT accumulation observed in the 16‐week‐old CD‐fed but not in HFD‐fed *Rptor*_*ob*_^*−/−*^ mice is likely due to other factors (e.g., diet, metabolic hormones, or systemic energy status) and not the expansion or lipolysis of preexisting bone marrow adipocytes (BMAds).

Intriguingly, the significant expansion of BMAds in the CD‐fed female *Rptor*_*ob*_^*−/−*^ mice coincided with a calorie‐deficient state, in that their energy intake was substantially lower than their energy expenditure (Figure 4*G*). Expansion of BMAds has been reported in response to changes in nutrient availability including starvation (reviewed in Devlin^(^ ^63^ ^)^), leptin deficiency,^(^ ^64^ ^)^ dietary restriction (DR),^(^ ^65^ ^)^ and HFD feeding^(^ ^43^, ^49^ ^)^ in mice, and also in human with anorexia nervosa patients.^(^ ^66^ ^)^ BMAT expansion, under restricted dietary conditions, was shown to be responsible for elevated circulating adiponectin levels that promote systemic improvements in glucose metabolism^(^ ^66^, ^67^, ^68^ ^)^ and, as such, elevated serum adiponectin levels could contribute to the improved metabolic phenotype of *Rptor*_*ob*_^*−/−*^ mice. DR has been shown to extend lifespan in several organisms^(^ ^69^ ^)^ and reduce or delay the development of age‐related diseases such as cardiovascular disease, cancer, metabolic diseases, and muscle atrophy.^(^ ^70^, ^71^, ^72^ ^)^ During DR, low nutrient and high AMP/ATP ratios activate AMPK, which inhibits mTORC1 by activating TSC2,^(^ ^12^ ^)^ a negative regulator of mTORC1, and/or by phosphorylating RAPTOR.^(^ ^13^ ^)^ Consistent with this view, pharmaceutical inhibition of TOR signaling (via rapamycin treatment) extends lifespan^(^ ^17^, ^73^, ^74^ ^)^ and genetic ablation of S6K1 in mice (S6K1^−/−^) leads to a gene expression profile similar to that of DR mice. Of note, female S6K1^−/−^ mice are long‐lived and have a reduced incidence of age‐related diseases.^(^ ^20^ ^)^ Concordance in the phenotypes of calorie‐restricted mice and *Rptor*_*ob*_^*−/−*^ mice (i.e., hypoleptinemia, hypoinsulinemia, high corticosterone, and an increase in ketone bodies) suggests that loss of mTORC1 function in OBs mimics a starvation response. Furthermore, a reduction in bone mass and an expansion of BMAT in the female *Rptor*_*ob*_^*−/−*^ mice combined with elevated circulating adiponectin levels, as evident in this study, suggest that suppression of mTORC1 function in OBs triggers a “starvation response” in the bone which leads to starvation‐induced BMAT formation and physiological adaptation to starvation.

### Sex‐dimorphism effects of OB mTORC1 inactivation

In contrast to CD‐fed male *Rptor*_*ob*_^*−/−*^ mice,^(^ ^27^ ^)^ an improvement in glucose tolerance and reduction in % fat mass were not observed in female CD‐fed *Rptor*_*ob*_^*−/−*^ mice (Table 2). We speculate that this may be attributable to sex asymmetry and the effect of sex hormones on metabolism (reviewed in Mauvais‐Jarvis^(^ ^75^ ^)^). Females resist the loss of energy stores better than males, which may also contribute to the sex‐specific differences observed in *Rptor*_*ob*_^*−/−*^ mice. Indeed, sex‐specific differences in the ability to resist loss of fat storage are known to occur in other stains of mice.^(^ ^76^, ^77^ ^)^ In female CD‐fed *Rptor*_*ob*_^*−/−*^ mice, their unaltered energy expenditure, despite a lower food intake (either absolute or adjusted to their body size), suggests that they are in a caloric deficit. Recent evidence suggests skeletally‐derived lipocalin (LCN2) can suppress food intake^(^ ^78^ ^)^; however, we found no changes in circulating LCN2 levels in female *Rptor*_*ob*_^*−/−*^ mice compared to controls (data not shown). In contrast, circulating leptin levels were significantly decreased in female CD‐fed *Rptor*_*ob*_^*−/−*^ mice, despite their lower food intake. We postulate that the hypoleptinemia observed in *Rptor*_*ob*_^*−/−*^ mice is a consequence of their low adiposity and negative energy balance (due to their lower dietary intake); however, further studies are required to determine if other appetite‐regulating hormones (i.e., ghrelin, neuropeptide Y, peptide YY, and other gut hormones) could be playing a role.

In recent years, the effects of mTORC1 perturbation have been examined in several different metabolic tissues including WAT, skeletal muscle, and liver, with each revealing a distinctive role for mTORC1 signaling in the tissue itself, and on global metabolism as a whole. For example, HFD‐fed mice with hepatic knockdown of S6K1/2 have improved systemic insulin sensitivity and glucose tolerance, and are protected against hepatic steatosis.^(^ ^79^ ^)^ In contrast, mice with deletion of *Rptor* in WAT suffer from severe hepatic steatosis despite being protected against diet‐induced obesity (via increased energy expenditure).^(^ ^80^, ^81^ ^)^ Similarly, inactivation of mTORC1 in skeletal muscle causes resistance to HFD‐induced metabolic dysfunction and improved glucose tolerance but has no effect on insulin sensitivity.^(^ ^82^ ^)^ Furthermore, systemic inhibition of mTORC1 signaling by rapamycin administration^(^ ^83^ ^)^ or deletion of S6K1^(^ ^19^ ^)^ results in mice that are resistant to weight gain due to elevated lipolysis and metabolic rate. Interestingly, despite the reported mechanistic differences between these models, one phenotype that is shared among these systemic and tissue‐specific mTORC1 knockout (KO) models is that they are resistant to HFD‐induced weight gain. In the current study, suppression of mTORC1 in OBs was also associated with resistance to HFD‐induced weight gain, consistent with previous mTORC1 KO models. Although the mechanism underlying obesity‐resistant phenotype of HFD‐fed *Rptor*_*ob*_^*−/−*^ mice requires further investigation, the mechanism is likely to involve the upregulation of *Ucp1* expression in the WAT depots of *Rptor*_*ob*_^*−/−*^ mice. Similar to our previous findings in male mice,^(^ ^27^ ^)^ upregulation of UCP1 in WAT was independent of previously reported browning agents including FGF21,^(^ ^84^ ^)^ irisin,^(^ ^85^ ^)^ sclerostin,^(^ ^86^ ^)^ and BMP7^(^ ^87^ ^)^ (Supplementary Figure S2). It is also interesting to note that the upregulation of UCP1 in *Rptor*_*ob*_^*−/−*^ mice appears to be depot‐specific, with a marked induction only observed in the iWAT, whereas upregulation of *Ucp1* mRNA, but not protein, was observed in gonadal WAT (gWAT). Furthermore, even though BMAT in the proximal tibial (which are juxtaposed to trabecular bone) has been reported to express markers of brown fat,^(^ ^88^, ^89^ ^)^ we were unable to detect *Ucp1* transcripts in the bone of *Rptor*_*ob*_^*−/−*^ mice (data not shown). Collectively, these results suggest that the mechanism(s) underlying this increased browning of white fat in *Rptor*_*ob*_^*−/−*^ mice could be due to systemic metabolic alterations or potentially occur in an endocrine, not paracrine, manner. Further studies interrogating RNA sequencing data to identify translated genes that encode secreted proteins and nontargeted metabolomic profiling of serum,^(^ ^90^ ^)^ involving the use of nuclear magnetic resonance mass spectrometry or complementary technologies, would be helpful to identify novel factor(s) responsible for the metabolic phenotype of *Rptor*_*ob*_^*−/−*^ mice.

One potential limitation of this study is the use of wild‐type littermates as the control group. Several previous studies have reported Osterix promoter activity^(^ ^91^, ^92^ ^)^ and Cre recombinase activity (in tTA:osx:cre mice^(^ ^93^ ^)^), in tissues other than bone. These observations, in addition to the well‐documented skeletal phenotype of tTA:Osx:cre mice^(^ ^94^, ^95^, ^96^ ^)^ has raised doubts as to the use of wild‐type mice as controls. To mitigate this issue, in our previous publication we compared the metabolic phenotype of both male and female tTA:Osx:cre mice with age‐ and sex‐matched wild‐type littermates. No differences in fasting glucose levels, glucose tolerance, insulin sensitivity, and serum osteocalcin (total and undercarboxylated) levels were observed.^(^ ^27^ ^)^ In regard to the potential influence of the tTA:Osx:cre transgene on bone biology and glucose homeostasis, most differences between wild‐type and tTA:Osx:cre mice are evident in early postnatal mice; that is, less than 6 weeks of age,^(^ ^94^, ^95^, ^96^ ^)^ and their bone mass (both cortical and trabecular) normalizes between 6 and 12 weeks of age.^(^ ^96^ ^)^ Because our studies were performed at 8 and 16 weeks of age, we believe that the influence of the tTA:Osx:cre transgene in bone, and hence glucose homeostasis, will be negligible.

Lineage tracing studies in tTA:Osx:cre mice have also revealed potential transgene expression in bone marrow adipocytes^(^ ^93^, ^97^ ^)^ suggesting altered mTORC1 signaling in BMAT may contribute to the metabolic phenotype of *Rptor*_*ob*_^*−/−*^ mice. Indeed, evidence from other studies suggests mTORC1 plays an important role in adipose tissue in the control of glucose homeostasis.^(^ ^80^, ^81^ ^)^ However, no Cre activity in the peripheral adipose tissue was reported in any of the lineage tracing studies using tTA:Osx:cre mice and thus any adipose‐specific contribution to the metabolic phenotype of *Rptor*_*ob*_^*−/−*^ mice is limited to BMAT. It should be noted that an improvement in glucose disposal and insulin sensitivity in female HFD‐fed *Rptor*_*ob*_^*−/−*^ mice (Figure 5) occurred without an increase in BMAT. Further investigation is required to determine the role of mTORC1 in BMAT in glucose homeostasis and how this potentially contributes to the metabolic phenotype of *Rptor*_*ob*_^*−/−*^ mice.

In summary, inactivation of bone‐specific mTORC1 in female mice lead to low bone mass at younger ages, which was attenuated by 16 weeks of age. Importantly, these mice are protected from HFD‐induced marrow adiposity expansion, obesity, and insulin resistance, suggesting an important role for the mTORC1 pathway in the skeletal regulation of glucose metabolism, and providing strong evidence to support the concept that dysregulated insulin signaling in OBs has systemic effects on glucoregulation and energy homeostasis. These studies highlight the beneficial systemic consequences of reducing mTORC1 function in OBs, under nutrient excess, revealing skeletal mTORC1 as a potential target for anti‐obesity and diabetes drugs.

## Author Contributions

**Pawanrat Tangseefa:** Data curation; formal analysis; writing‐original draft; writing‐review & editing. **Sally Martin:** Conceptualization; data curation; formal analysis; writing‐review & editing. **Agnieszka Arthur:** Formal analysis; writing‐review & editing. **Vasilios Panagopoulos:** Writing‐review & editing. **Amanda Page:** Writing‐review & editing. **Gary Wittert:** Writing‐review & editing. **Christopher Proud:** Conceptualization; funding acquisition; writing‐review & editing. **Stephen Fitter:** Conceptualization; data curation; formal analysis; funding acquisition; supervision; writing‐original draft; writing‐review & editing. **Andrew Zannettino:** Conceptualization; data curation; formal analysis; funding acquisition; supervision; writing‐review & editing.

## Conflicts of Interest

The authors declare no conflicts of interest.

### Peer Review

The peer review history for this article is available at https://publons.com/publon/10.1002/jbm4.10486.

## Supporting information


**Supplementary Figure S1** Marrow adiposity was unchanged in 4‐week‐old female CD‐fed Rptorob^−/−^ mice. (A) Representative H&E‐stained sections of the proximal tibia in 4‐week‐old CD‐fed mice. Scale bar = 500 μm. (B) Percentage area occupied by adipocytes per marrow area (%Ad.Ar/Ma.Ar). (C) Average adipocyte size (Ad.Ar/N.Ad). (D) Numbers of adipocytes per marrow area (N.Ad/Ma.Ar). All panels except A: data are presented as presented as mean ± SEM from *n* = 3/genotype. Student *t* test.

**Supplementary Figure S2** Browning of iWAT in HFD‐fed Rptorob^−/−^ mice occurs independent of known browning inducers. (A) Fgf21 gene expression, normalized to β‐actin in liver. (B) *Fndc5* gene expression, normalized to β‐actin in muscle. (C) *Bmp7* and (D) *Sost* gene expression, normalized to β‐actin in bone samples. All panels: data are expressed as mean ± SEM from *n* = 4/genotype. Student *t* test.
