# Supplementary figures and images for: Deletion of Rptor in Preosteoblasts Reveals a Role for the Mammalian Target of Rapamycin Complex 1 (mTORC1) Complex in Dietary‐Induced Changes to Bone Mass and Glucose Homeostasis in Female Mice
Source: JBMR Plus. 2021 Mar 24;5(5):e10486. doi: 10.1002/jbm4.10486 (PMC8101617; doi:10.1002/jbm4.10486)

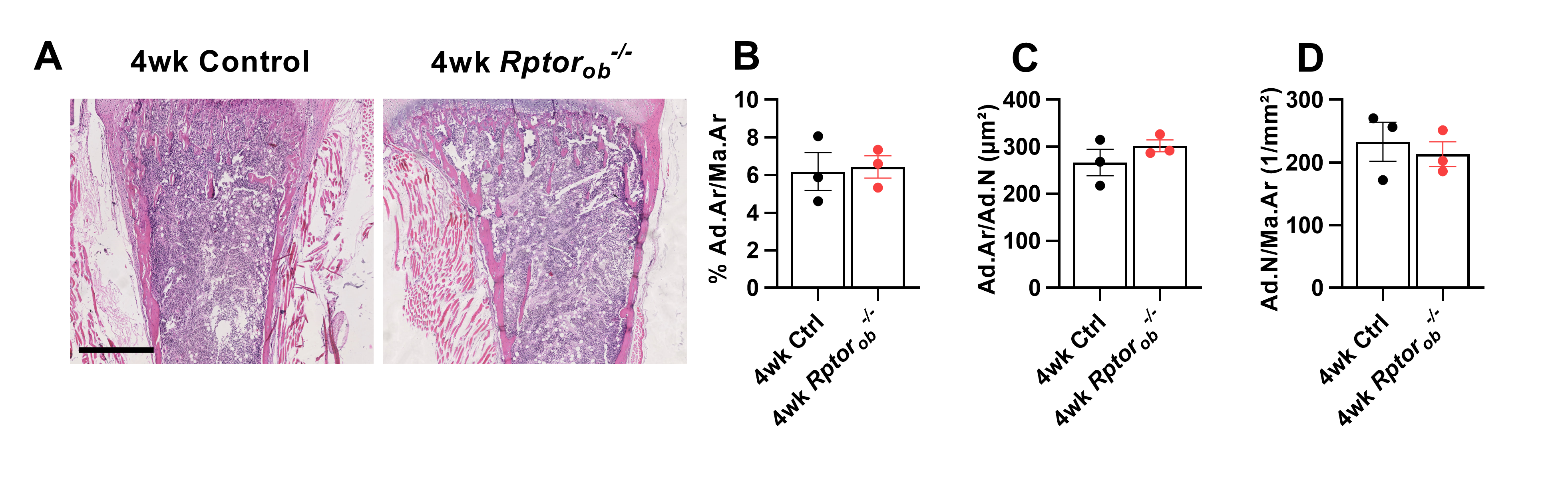

Supplement: Supplementary file 1 — Supplementary Figure S1 Marrow adiposity was unchanged in 4‐week‐old female CD‐fed Rptorob−/− mice. (A) Representative H&E‐stained sections of the proximal tibia in 4‐week‐old CD‐fed mice. Scale bar = 500 μm. (B) Percentage area occupied by adipocytes per marrow area (%Ad.Ar/Ma.Ar). (C) Average adipocyte size (Ad.Ar/N.Ad). (D) Numbers of adipocytes per marrow area (N.Ad/Ma.Ar). All panels except A: data are presented as presented as mean ± SEM from n = 3/genotype. Student t test. [file JBM4-5-e10486-s002.tif]

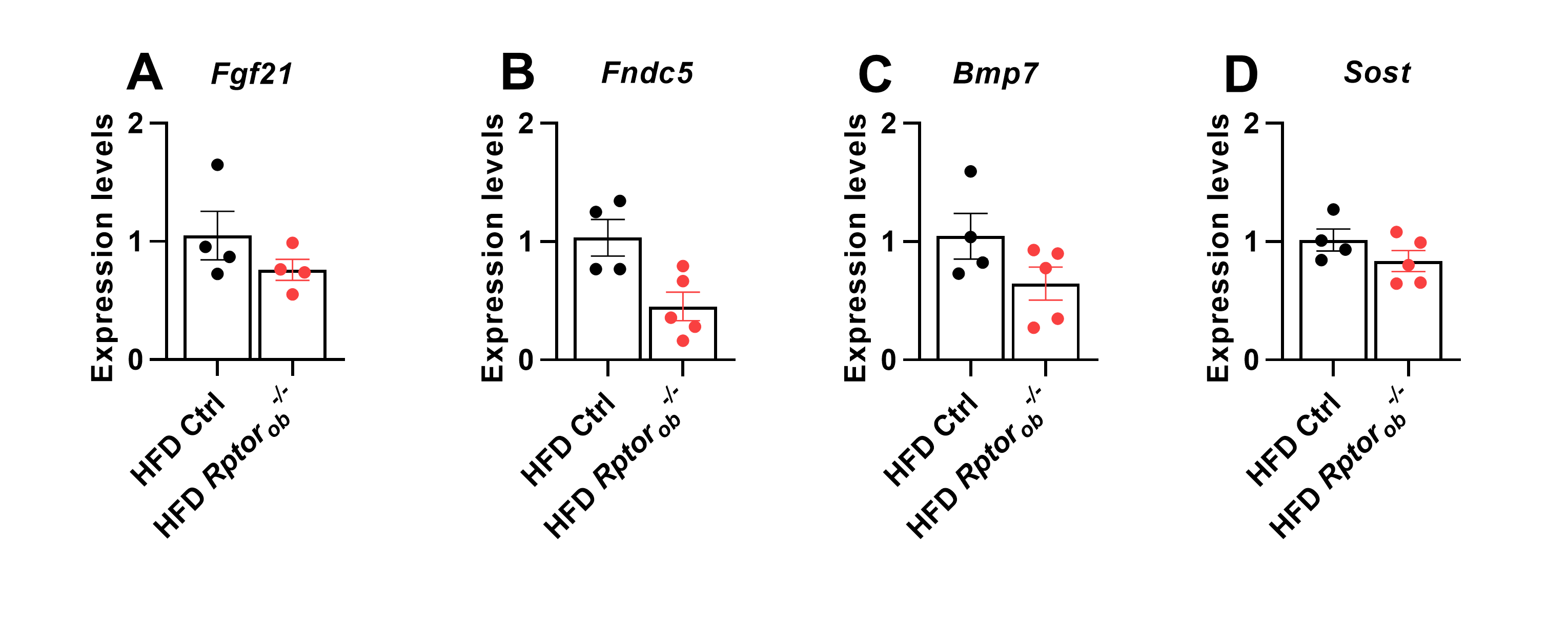

Supplement: Supplementary file 2 — Supplementary Figure S2 Browning of iWAT in HFD‐fed Rptorob−/− mice occurs independent of known browning inducers. (A) Fgf21 gene expression, normalized to β‐actin in liver. (B) Fndc5 gene expression, normalized to β‐actin in muscle. (C) Bmp7 and (D) Sost gene expression, normalized to β‐actin in bone samples. All panels: data are expressed as mean ± SEM from n = 4/genotype. Student t test. [file JBM4-5-e10486-s001.tif]
